# Role of ChREBP–PPARα–FGF21 Axis in Metabolic Dysfunction of MASLD

**DOI:** 10.3390/ijms262311425

**Published:** 2025-11-26

**Authors:** Karina Mireya Palacios Girón, Zamira Helena Hernandez Nazara, Montserrat Maldonado-González, Erika Martínez-López, Martha P. Sánchez Muñoz, Carlos Alfredo Bautista López, Ma. Soledad Aldana Aguiñaga, Jose Alfredo Dominguez-Rosales, Belinda Vargas-Guerrero, Bertha Ruíz-Madrigal

**Affiliations:** 1 Programa de Doctorado en Ciencias en Biología Molecular en Medicina, Departamento de Biología Molecular y Genómica, Centro Universitario de Ciencias de la Salud, Universidad de Guadalajara, Guadalajara C.P. 44340, Mexico; mireya.palacios@alumnos.udg.mx; 2 Instituto de Investigación en Enfermedades Crónico Degenerativas (IECD), Departamento de Biología Molecular y Genómica, Centro Universitario de Ciencias de la Salud, Universidad de Guadalajara, Guadalajara C.P. 44340, Mexico; zamira.hernandez@academicos.udg.mx (Z.H.H.N.); belinda.vargas@academicos.udg.mx (B.V.-G.); 3 Centro de Investigación en Enfermedades Infectocontagiosas (CIEIC), Departamento de Microbiología y Patología, Centro Universitario de Ciencias de la Salud, Universidad de Guadalajara, Guadalajara C.P. 44340, Mexico; montserrat.maldonado@academicos.udg.mx; 4 Instituto de Nutrigenética y Nutrigenómica Traslacional (INNUGET), Departamento de Biología Molecular y Genómica, Centro Universitario de Ciencias de la Salud, Universidad de Guadalajara, Guadalajara C.P. 44340, Mexico; erika.martinez@academicos.udg.mx; 5 Unidad de Cirugía Bariátrica y Metabólica, Nuevo Hospital Civil de Guadalajara Dr. Juan I. Menchaca, Guadalajara C.P. 44340, Mexico; cirugiapatriciasanchez@gmail.com (M.P.S.M.); soledaldana@gmail.com (M.S.A.A.); 6 Servicio de Cirugía General, Nuevo Hospital Civil de Guadalajara Dr. Juan I. Menchaca, Guadalajara C.P. 44340, Mexico; alfredo.bautista@academicos.udg.mx

**Keywords:** Metabolically Dysfunctional-Associated Steatotic Liver Disease (MASLD), MLX interacting protein-like (*MLXIPL*), Carbohydrate Response Element Binding Protein (ChREBP), Peroxisome Proliferator-Activated Receptor Alpha (*PPARA*), Fibroblast Growth Factor 21 (*FGF21*)

## Abstract

Metabolically Dysfunctional-Associated Steatotic Liver Disease (MASLD) affects both metabolically healthy obese (MHO) individuals and metabolically unhealthy lean (MUL) individuals. Key genes linked to liver dysfunction, such as *MLXIPL*, *PPARA*, and *FGF21*, are under-researched in humans. We aimed to evaluate the ChREBP–PPARα–FGF21 axis in relation to metabolic liver dysfunction in MHO and MUL individuals. Liver histopathology, biochemical data, anthropometric measurements, mRNA expression as analyzed by qPCR, and heatmap visualization were utilized to identify relationships among variables and discern gene expression patterns. ChREBP–PPARA–FGF21 genes were analyzed in liver, subcutaneous (SAT), and omental adipose tissue (OAT) biopsies from 55 subjects, including metabolically unhealthy obese (MUO), MHO, MUL, and metabolically healthy lean (MHL) subjects as controls. The MHL, MUL, MHO, and MUO groups showed a gradual increase in liver PPARα, with a downward trend in ChREBPβ levels in SAT (*p* < 0.05). Liver ChREBPβ positively correlated with insulin resistance and *FGF21*. Levels of OAT ChREBPβ showed a negative correlation with anthropometric measurements and serum insulin levels. These findings suggest coordinated regulation under metabolic stress. Increased *FGF21* expression in the MUL and MUO groups may aid as a metabolic biomarker of impaired energy homeostasis and compensatory hepatic response.

## 1. Introduction

Metabolic Dysfunction-Associated Steatotic Liver Disease (MASLD) is an increasingly prevalent disease associated with obesity [1]. Besides, obesity is part of metabolic syndrome (MetS); obesity is a disorder that leads to years lived with disability that requires medical treatment, causes potentially life-threatening complications, and contributes to the primary causes of mortality worldwide (e.g., type 2 diabetes (T2D), cardiovascular disease (CAD), kidney failure, certain cancers, and mental disorders).

Recently, the term MASLD has been adopted for individuals with steatosis, one or more cardiometabolic risk factors, and the absence of harmful alcohol intake [2]. While several authors have identified individuals with metabolically healthy obesity (MHO), defined by the presence of ≤2 components of MetS [3], lean individuals with MetS are considered metabolically unhealthy (MUL) [4].

On the other hand, there is evidence in murine models indicating a cross-interaction between the following genes: Fibroblast Growth Factor 21 (*FGF21*); Peroxisome Proliferator-Activated Receptor Alpha (*PPARA*), which regulates lipid metabolism and energy expenditure; and the MLX interacting protein-like (*MLXIPL*), also known as carbohydrate-responsive element-binding protein (ChREBP), encoding a protein of the same name and its isoforms ChREBPα and -β, which are the link to de novo lipogenesis in response to carbohydrates from the diet [5]. ChREBP/PPARα cross-talk is necessary to activate *FGF21*, preventing fatty infiltration in the liver, which is considered beneficial [6,7,8]. However, the characteristics of liver molecular dysfunction have been investigated less in humans with MUL and MHO conditions.

Our focus is on enhancing the understanding of variability in hepatic metabolic dysfunction. Identifying all phenotypes is crucial for providing precision medicine by mapping the entire spectrum of diseases. The accurate identification of individuals with MHO, MUL, metabolically unhealthy obese (MUO), and metabolically healthy lean (MHL) conditions is essential for proper diagnosis, treatment, and personalized medicine. Therefore, in this study, we aim to characterize the presence of MASLD by examining histopathological changes in the liver, as well as biochemical and anthropometric variables. We will also assess the expression of the *ChREBP*–*PPARα*–*FGF21* axis through simultaneous biopsies of subcutaneous adipose tissue (SAT), omental adipose tissue (OAT), and liver in the study groups. We hypothesize that differential expression of the *ChREBP*–*PPARα*–*FGF21* axis across liver, subcutaneous, and omental adipose tissues could distinguish metabolic conditions (MHL, MHO, MUL, and MUO) and correlate with hepatic histopathological changes and biochemical indicators of metabolic dysfunction, thereby contributing to the variability and progression of MASLD.

## 2. Results

### 2.1. Characteristics of the Study Population

The biochemical, anthropometric, and histopathological data are presented in Table 1, and the grouping characteristics are in accordance with the criteria of Smith GI et al. [4]. Weight, BMI, waist circumference, and body fat % data determine the differences between individuals with and without obesity. Because the blood glucose levels were below the diagnostic threshold for T2D in each case in this study, blood insulin levels and a >2.5 HOMA IR index value determine the differences between the metabolically healthy and unhealthy groups [9].

Differences in AST and ALT levels indicate individuals presenting liver damage, which aligns with the significance of 85% of MASLD being detected in individuals with MUO vs. MHL (X^2^ = 26.5, *p* < 0.001) and MUO vs. MUL (X^2^ = 7.8, *p* < 0.01) and 62% of MASLD being detected in individuals with MHO vs. MHL (X^2^ = 10, *p* < 0.01). Nonsignificant differences are found in other MASLD percentages, such as individuals with MHO and MUO, despite the MUL group including all MASLD in lean individuals. It is worth mentioning that the levels of liver enzymes, including GGT, tend to increase in metabolically unhealthy patients regardless of their obesity status. However, the increases in AST and ALT are notably significant in the MUO group (*p* < 0.05). The liver enzyme values do not reach or exceed threshold levels, as the selection criteria exclude patients diagnosed with acute hepatitis.

The commonly accepted threshold for high-sensitivity C-reactive protein (hs-CRP) is 3 mg/L; therefore, values above this threshold (>3 mg/L) are outside the normal range. Consequently, the average hs-CRP levels observed in MHO or MUO patients are considered high, indicating nonspecific inflammation, even though no significant differences were found between groups.

Lipid values do not present significant differences between groups; however, the qualitative threshold values, specifically, TG levels > 150 mg/dL, total cholesterol levels > 200 mg/dL, and HDL-c levels < 40 mg/dL, distinguish the metabolically unhealthy group from the healthy one. There are no significant differences in age and sex between the study groups, with most of the participants being women and the average age being ~37 years old.

### 2.2. qPCR Gene Expression Analysis of mRNA

**Figure 1:**
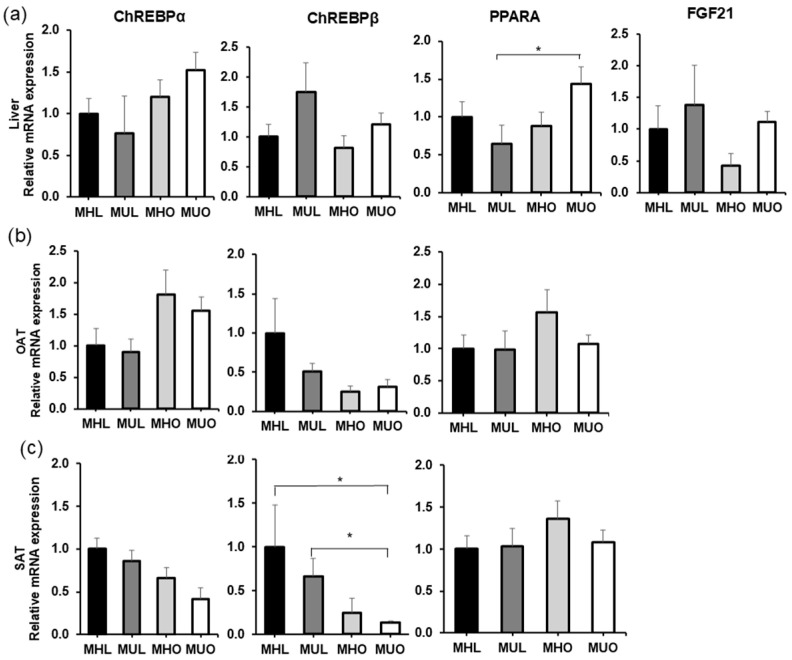
ChREBP isoforms, *PPARA*, and *FGF21* mRNA expression levels were determined by qPCR in simultaneous biopsy samples from participants classified as MHL, MUL, MHO, and MUO. Relative mRNA expression was analyzed in (**a**) liver, (**b**) omental adipose tissue (OAT), and (**c**) subcutaneous adipose tissue (SAT). Expression levels were normalized to *POLR2A*. Data represent the mean ± SEM (n = 12 for MHL, n = 6 for MUL, n = 8 for MHO, and n = 29 for MUO), obtained from triplicate assays. Statistical differences were assessed using ANOVA or the Kruskal–Wallis test as appropriate, and significant differences are indicated by * (*p* < 0.05).

In liver biopsies, mRNA expression levels of the gene isoforms ChREBPα, ChREBPβ, *PPARA*, and *FGF21* were detected (Figure 1a). There was an upward trend in the liver ChREBPα and PPARA mRNA levels in lean individuals compared to those in the MHO and MUO groups. The MUL group showed a 75% increase in liver ChREBPβ expression, while MHO showed 60% less *FGF21* expression compared to the controls MHL and MUL. However, no statistically significant differences were revealed in the above findings. A 79% difference is observed in the expression levels of PPARA mRNA between the MUL and MUO groups, which is statistically significant (*p* < 0.05).

We did not find expression of *FGF21* in adipose tissues (Figure 1b,c). The data were unreliable because Cq values of 35 and above indicate single-copy reactions or noise in a qPCR assay. The expression of the genes of interest did not differ significantly in OAT (Figure 1b). An inverse gradient of mRNA levels of the *PPARA* and ChREBPα isoforms is observed compared to the ChREBPβ isoform across lean and obese individuals.

In the SAT, we observed a tendency for lower expression of the ChREBPα isoform in lean individuals compared to obese individuals, reaching up to 58% between the MHL and MUO groups. However, this was not significant. The effect is more pronounced in the ChREBPβ isoform, reaching significance between MHL vs. MHO and MUL vs. MUO (*p* < 0.05). Nonsignificant differences in *PPARA* expression values were found in SAT (Figure 1c).

#### 2.2.1. Correlation Analysis

The correlation analysis of liver genes with clinical, anthropometric, and biochemical parameters revealed the following results: *PPARA* showed a moderate correlation with liver ChREBPα expression (rs = 0.4), *FGF21* (rs = 0.45), and ALT (rs = 0.45). The HOMA-IR index (rs = 0.36), GGT serum levels (rs = 0.34), *FGF21* expression (rs = 0.57), and liver ChREBPα (rs = 0.49) showed a weak-to-strong correlation with liver ChREBPβ mRNA (*p* < 0.05); thus, it was considered biologically relevant due to the existing biological plausibility supporting the association.

Significantly, SAT ChREBPβ moderately correlated with SAT ChREBPα (rs = 0.45) and OAT ChREBPβ (rs = 0.46) expression, and moderately to strongly negatively correlated with anthropometric variables, IMC (rs = −0.47), waist circumference (rs = −0.48), weight (rs = −0.49), and body fat % (rs = −0.53) (*p* < 0.05).

**Figure 2:**
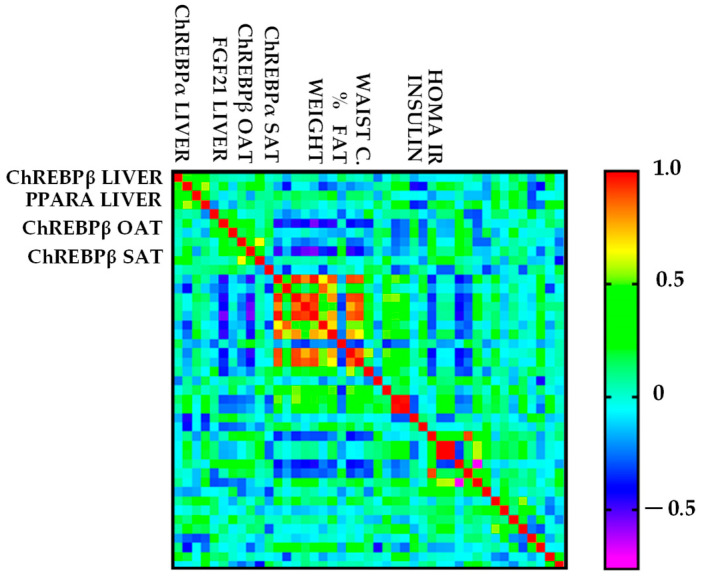
Correlogram showing the Spearman correlation coefficients (rs) among clinical, anthropometric, and biochemical parameters and target gene expression levels in the total sample (n = 55). The color scale represents the direction and strength of the correlations (violet: negative; red: positive). Only statistically significant correlations (*p* < 0.05) are shown. The main relationships are described in detail in the text.

Additionally, anthropometric variables, including weight (rs = −0.47), IMC (rs = −0.46), body fat % (rs = −0.4), waist circumference (rs = −0.42), and serum insulin (rs = −0.31), showed a weak to moderately negative correlation with OAT ChREBPβ. These findings highlight the medium relationships between gene-target expressions and metabolic dysfunction, with positive correlations in liver mRNA expression and negative correlations in adipose tissue mRNA expression (Figure 2).

#### 2.2.2. Heatmap Analysis

Specifically, the high expression of liver *PPARA* in MUO vs. MUL individuals and the lower SAT ChREBPβ in lean individuals vs. MUO showed significant differences (*p* < 0.05) (Figure 1), indicating that these target genes play a role in the conditions examined in this study.

**Figure 3:**
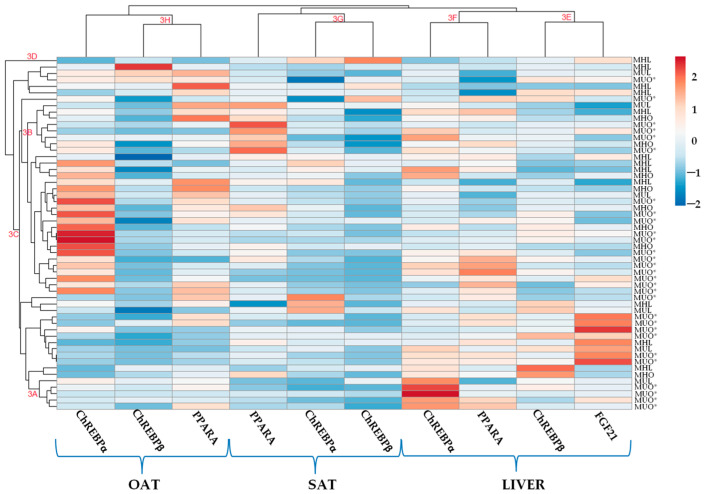
Heatmap showing hierarchical clustering of differentially expressed genes (ChREBP isoforms, *PPARA*, and *FGF21*) across liver, omental adipose tissue (OAT), and subcutaneous adipose tissue (SAT) samples (columns) from participants with MHL, MUL, MHO, and MUO* conditions (rows; n = 55). Clustering branches (A–H) represent distinct expression profiles. The color scale indicates relative expression levels (log_2_ fold change). The color scale denotes relative expression levels, ranging from blue (low expression) to red (high expression).

Furthermore, a 2D heatmap graphic was generated to visualize the available clustering distances and density distributions (Figure 3). The hierarchical clustering patterns in the rows display the distribution density of patients based on the similarity of their gene target expression, showing heterogeneity mainly in the MUO* condition. However, in the rows, ChREBPα expression is upregulated solely in the MUO* condition in the liver (3A in Figure 3) and in the OAT, including most MUO* individuals. Therefore, MHL and MHO individuals are mainly clustered differently in the medium (3B in Figure 3) and highest branches (3C in Figure 3), including a simplicifolious MHL individual (3D in Figure 3). These groups are highly differentiated because the top of the dendrogram heatmap reveals the main branches, as indicated. The clustering patterns indicate variations in gene expression across tissues between different condition groups. While metabolically healthy individuals cluster, unhealthy persons form more widely separated branches.

The clustering patterns of target gene expression across different tissues, presented in the columns (Figure 3), demonstrate a primary relationship within intra-organ target gene expression. The heatmap and dendrogram show the clustering of samples with similar gene expression and the clustering of genes with comparable expression patterns. In this study, two clusters of liver expression of ChREBPβ–*FGF21* (3E in Figure 3) and ChREBPα–*PPARA* (3F in Figure 3) were identified. Similarly, within the same branch, the expression of ChREBPα–ChREBPβ (3G in Figure 3) in SAT is further distinct from that of *PPARA*. The cluster of ChREBPβ–*PPARA* (3H in Figure 3) exhibits an inverse connection to ChREBPα in OAT. These data are consistent with the histograms in Figure 1 and refer to the uniformity, accuracy, and reliability of the same information analyzed by two different methods.

#### 2.2.3. Liver Heatmap Analysis

**Figure 4:**
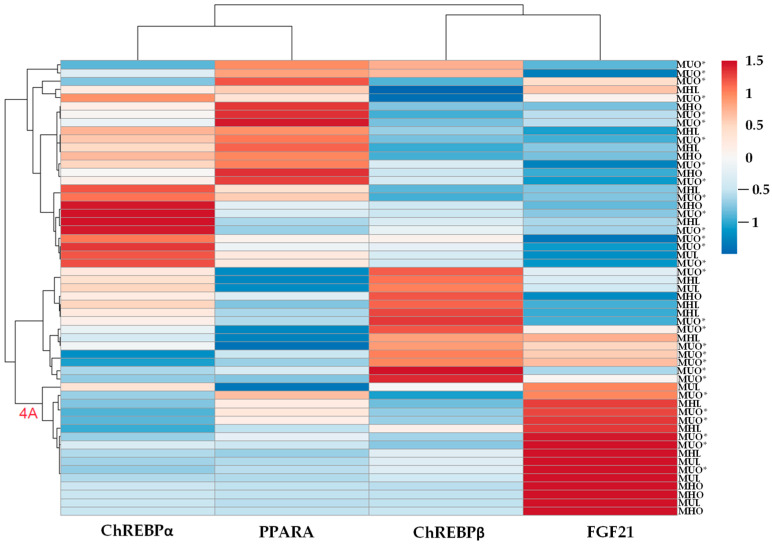
Heatmap illustrating hierarchical clustering of hepatic expression levels of ChREBP isoforms, *PPARA*, and *FGF21* (columns) across individuals classified as MHL, MUL, MHO, and MUO* (rows; total n = 55). Expression values are shown as log_2_ fold change. The analysis was performed using ClustVis 2.0, applying hierarchical clustering with Euclidean distance and average linkage. Branch 4A corresponds to a subgroup displaying increased *FGF21* expression. The color scale denotes relative expression levels, ranging from blue (low expression) to red (high expression).

In general, the differential expression of the heatmap for the liver does not show differential expression (Figure 4), so it could be more complex; for instance, if the patients are very mixed in the branches of the dendrogram, it could indicate that the variability in gene expression is not strongly related to the state of the disease conditions studied. However, it is essential to highlight that the MUL group is observed to be clustered mainly in the secondary branch, with the highest expression of *FGF21* in rows and downregulation of *PPARA*, primarily in lean individuals (4A in Figure 4). Similar to Figure 1 and Figure 3, this confirms that patients with upregulated *PPARA* expression are closer to the upregulated expression of the ChREBPα cluster, and the ChREBPβ cluster nearly matches the upregulation of *FGF21* in the columns.

## 3. Discussion

There is significant debate regarding both the existence and the diagnostic criteria for MHO and MUL individuals [4]. In this study, we identified 8 MHO and 6 MUL conditions from a cohort of 55 individuals (primarily women, 87%). Nevertheless, it is necessary to differentiate between the MHO group, as their medical approach cannot be generalized and treated as an MUO condition [10]. Obesity itself has been considered an abnormal condition since 1948 [11]. Obesity has side implications in addition to metabolic ones, including a higher risk of renal failure [12], cancer [13], and other typical conditions like osteoarthritis [14], sleep apnea [15], psychosocial issues [16], and a general decline in quality of life. At the same time, MHO is a transient state that evolves into MUO. According to different studies, the transition to metabolic problems between 4 and 20 years is highly variable [4]. Elías-López et al. found that 40.6% of the Mexican population (mainly females aged 40 years) transitioned from the MHO to the MUO group over a three-year period [17].

On the other hand, the MUL condition lies in their common underdiagnosis and the fact that their progression to more serious metabolic complications is often worse than that of MHO individuals [18]. These events are closely related to the controversial concept of the “obesity paradox” [19]. Even the presence of MASLD in lean individuals has been reported to result in the most severe outcomes [20,21,22]. Nonetheless, for other authors, the progression to more severe complications for the MUL group or the occurrence of MASLD in lean persons remains ambiguous [23,24]. This condition is notably observed in Asians [25,26].

This study required an explanation for the setting of TG cut-off levels at <95 mg/dL to distinguish between healthy and unhealthy participants. Nevertheless, the average readings for lean and obese healthy patient groups exceeded the cut-off point. Cardiovascular disease, MetS, and hypertriglyceridemia [27,28] are all highly prevalent in the Mexican population under study [29]. Therefore, hypertriglyceridemia is frequently the only metabolic risk factor in the healthy population, regardless of ethnicity [30]. The absence of difference in TG between the groups may also stem from the patients enrolled in this study having choledocholithiasis, a condition associated with high TG levels [31,32].

In this study, we adhered to the definition of Smith GI et al. and included the HOMA-IR index (>2.5). To group patients as metabolically unhealthy, more than two findings’ traits must be present [4]. Patients with diabetes were excluded, as insulin insensitivity is essential for identifying metabolic abnormalities and differentiating them from the metabolically healthy groups [33]. The need for evaluation beyond this criterion to detect metabolic changes in individuals that appear to be normoglycemic is highlighted by the fact that serum glucose levels were below the diagnostic threshold for diabetes mellitus in every instance. Hence, the MUO and MUL groups consist of patients with prediabetes. However, blood insulin levels are more effective in staging and grading than HOMA-IR because we found significant differences between healthy and unhealthy conditions. The above is consistent with previous studies [34]. Likewise, advanced glycation markers may help to diagnose these metabolic conditions and monitor their progression in individuals with MHO vs. MUO [35,36].

As with TG levels, hs-CRP levels did not differ significantly between groups. However, the mean values exceeded the metabolic risk threshold of >3 mg/dL in the MHL, MHO, and MUO groups. Interestingly, our data indicate that the average hs-PCR level > 5 mg/dL is present in obese individuals and the MASLD groups (MHO and MUO). Hence, it agrees with recent studies linking it to biopsy-proven MASLD mainly in obese individuals [37]. However, no substantial differences were observed in high hs-PCR levels between the MHO and MUO groups, which is consistent with previous studies [38]. However, these findings are controversial [39,40,41,42]. hs-PCR is an inflammatory marker of innate defense that can recruit the complement system and phagocytic cells produced in the liver. This marker has been demonstrated to have prognostic value for hepatic and metabolic complications. Contradictorily, average values associated with lower risk < 3 mg/dL are present in the MUL group, and values > 3 mg/dl are seen in the MHL group.

Although GGT serum values were not statistically significant, they were better at discriminating between unhealthy and healthy conditions than hs-CRP measurements in this study. GGT removes the gamma-glutamyl moiety, degrading molecules such as amino acids and proteins including glutathione. The standard reference range for blood levels is below 40 U/L. The GGT levels are slightly above normal limits in the MUL and MUO groups, as a result of the excluded patients with acute liver injury based on elevated transaminase enzyme levels despite this being a standard indicator of hepatobiliary disorders. Even in lean individuals, steatosis and liver dysfunction have historically been identified using GGT and TG levels [43,44,45].

GGT sensitively measures and determines the mortality rate [46,47,48,49] and an early onset marker associated with metabolic diseases [50,51,52]. GGT can determine metabolic changes and monitor medical interventions [53,54]. For instance, GGT is linked to small, dense, and low-density lipoproteins [55,56] and can identify metabolic alterations, such as postprandial hypertriglyceridemia [57].

Therefore, it would be advisable for researchers to expand the investigation and evaluate the utility of GGT, particularly in studies that use surrogate indices of MASLD, such as the visceral adiposity index (VAI) and fatty liver index (FLI), and lack a confirmed biopsy [42,43]. The above is also true in metabolically high-risk situations, such as pregnancy [58,59]. Hence, we need to conduct a more thorough investigation of GGT for this subgroup of MetS [34].

Similar to transaminase levels, primarily ALT was relevant in this investigation for differentiating between healthy and unhealthy individuals, with significant differences observed between MUO and MHL [60,61]. The above finding is consistent with an extensive series of studies that have utilized liver enzymes to characterize these groups [62,63,64]. It is worth noting that the concentrations of liver enzymes did not reach diagnostic levels for acute hepatitis, likely due to the stringent exclusion criteria applied.

It is worth mentioning that, unlike other studies, we were unable to distinguish between MHO and MUO using waist circumference as an indirect measure of visceral fat distribution in this investigation [65]. The degrees of steatosis found in descending order are present in the MUO > MHO > MUL groups and absent in MHL. At least in our study, a lean person with steatosis consistently exhibits MetS and correlates with a diagnosis of MASLD belonging to the MUL group. Therefore, it is necessary to consider the MUL clinical diagnosis in lean individuals with steatosis to identify genetic rather than environmental factors associated with obesity.

### 3.1. PPARA Expression

One of the most significant findings was an upward trend in liver *PPARA* mRNA levels in lean individuals compared to those in the MUL and MUO groups, with a difference in liver *PPARA* expression between MUL and MUO individuals. The aforementioned implies that it is one of the distinctive traits between the two conditions. The transcription factor *PPARA* is extensively studied due to its integrative role in mitochondrial fatty acid oxidation and the production of ketone bodies in the liver. Therefore, it is considered an important therapeutic target [66]. In murine models with *PPARA* deficiency, it is demonstrated that this factor is crucial for lipid storage and energy homeostasis, leading to steatosis [67]. In humans, *PPARA* gene expression negatively correlates with the progression of steatotic liver disease [68]. The above findings are consistent with the low levels of liver *PPARA* expression observed in the MUL group.

However, the 50% increase in hepatic expression in the MUO group is striking. Consequently, we might consider this to be a compensatory increase in the MUO group; *PPARA* expression could increase as part of an adaptive attempt to restore lipid and mitochondrial homeostasis. Furthermore, we have not found any literature reports comparing liver *PPARA* mRNA expression in humans under these clinical grouping conditions during fasting.

The levels of *PPARA* mRNA expression in MUO do not necessarily indicate an increase in function, since it depends on the final protein synthesis and ultimate protein function. The levels of *PPARA* mRNA could indicate an unsuccessful metabolic resolution mechanism, as they increase the rate of lipid oxidation, meaning our results contradict those reported in the literature.

Additionally, results from transcriptomics studies in chimeric mice with humanized livers indicate that *PPARA* plays a significant role in regulating hepatic lipid metabolism [69]. However, compared to murine hepatocytes, human hepatocytes show a more moderate effect of *PPARA* activation on the induction of target genes, making cross-species comparisons challenging.

On the one hand, *PPARA* supports the preservation of mitochondrial function, for instance, in deficient rodent models (PPARα−/−), resulting in a decrease in key genes of mitochondrial β-oxidation of fatty acids with the effects of heightened lipid droplets in the liver, with increased mitochondrial stress markers such as HSP60 and increased serum levels of TG and cholesterol [66]. Therefore, *PPARA* agonists could mitigate mechanisms of lipotoxicity and oxidative stress [70,71].

Conversely, in diabetic cardiomyopathy models, *PPARA* activation increases the expression of genes related to β-oxidation, which generates reactive oxygen species (ROS) and contributes to heart dysfunction and mitochondrial damage [67,71,72]. Furthermore, several *PPARA* agonist compounds have advanced clinical stages before being discontinued due to adverse effects or lack of efficacy, and specific *PPARA* ligands and agonists are not approved for general use. For example, dual PPARα and PPARγ agonists such as Muraglitazar were in Phase III clinical trials. However, increasing mortality and adverse consequences such as weight gain, edema, and significant adverse cardiovascular events led to the discontinuation of their development [73]. Similarly, another dual PPARα and PPARγ agonist, Tesaglitazar, completed several Phase III clinical trials, and its development was discontinued due to cardiac toxicity associated with mitochondrial dysfunction [74,75]. More recently, the study of Aleglitazar as a dual PPARα and PPARγ agonist for the treatment of T2D was also discontinued due to side effects [76].

In line with the above, the action of *PPARA* agonists may depend on prior activation of the pathway [77] and underlying cellular damage, primarily mitochondrial stress [78] and senescence [79]. If the cell has prior damage or the *PPARA* pathway is activated, the agonists are likely to only worsen hepatic, cardiac, or systemic metabolic dysfunction. Therefore, it is necessary to carefully discern which patients are suitable to receive this group of drugs.

Interestingly, a meta-analysis by Ru-Tao Lin et al. evaluated various treatments for different therapeutic goals related to MASLD, with a focus on target genes such as *PPARA*. Their findings suggest that recommendations for Pan-PPAR provide a practical therapeutic approach that improves glycemia and protects the liver in non-obese patients suffering from MASH, liver injury, and T2D [80]. The above findings are consistent with our observations, as individuals with the MUL condition exhibit low levels of *PPARA* expression, suggesting that PPAR agonists may be beneficial in this population. Likewise, challenges such as frequent side effects underscore the need to optimize and combine strategies to maximize its therapeutic potential, for example, by taking it alongside antioxidants.

### 3.2. MLXIPL Expression

In this study, the expression of messenger RNA of the two ChREBPα and -β isoforms of the *MLXIPL* gene exhibited differences in expression that could aid the dissection of the characteristic patterns between the groups studied. Both isoforms transactivate the same genomic programs, which include the glycolytic and de novo lipogenic pathways, as well as those encoding enzymes for the elongation of fatty acids in response to circulating and dietary carbohydrates. As a result, these isoforms play a significant role in regulating metabolic traits [81,82,83]. The proteins ChREBPα and ChREBPβ exhibit different potencies and regulatory dynamics because of their distinct regulatory roles, resulting in biological effects that are comparable yet complementary [84,85]. ChREBPβ serves as a highly active amplifier of the lipogenic and glucose-sensing gene programs initiated by ChREBPα [81].

As determined by in vitro and in vivo studies, the expression levels of ChREBPα and ChREBPβ in adipose tissue are lower in obese individuals than in non-obese individuals. In contrast, their expression is increased in the liver during obesity and metabolic alterations [81,86,87,88]. In addition, the decrease in both ChREBP isoforms correlates with reduced expression of other lipogenic genes, signifying a wider suppression of adipogenic and lipogenic pathways. Therefore, we do not have an explanation for the increase in ChREBPα within OAT tissue based on the degree of obesity and metabolic state in this study, although this finding is not significant. On the other hand, ChREBPα correlates positively with ChREBPβ in SAT.

Previous reports indicate that OAT has lower levels of leptin and GLUT4 mRNA, displays less efficient insulin signaling, and exhibits a more pro-inflammatory profile. In contrast, SAT presents higher leptin and GLUT4 mRNA, more efficient insulin signaling, and browning [89,90,91,92,93]. Obesity exacerbates these differences, with OAT playing a central role in metabolic complications, while SAT may retain some protective features if its adaptive capacity is maintained. Together, our findings and the data from the literature suggest that lower expression of ChREBPβ in adipose tissue is associated with reduced insulin sensitivity and impaired glucose tolerance. The above indicates that it is a response to low Glut4 levels and low glucose cellular entry, with or without obesity, and is significantly lower in SAT, contrasting with its upregulation in the liver, which highlights tissue-specific regulation of metabolic status. The negative correlations in our findings between adipose tissue ChREBPβ isoform expression, anthropometric measurements, and reduced insulin levels align with the existing literature [87].

In the liver, ChREBPα exhibits an upward trend from lean individuals to those with MHO, particularly in the MUO group, although this difference does not reach statistical significance. Some studies report that ChREBPα is more abundant under normal conditions; if there is an increase in its expression in the liver of individuals with obesity, it is not dramatic [94,95]. Our findings are in agreement with those of the last study. Other studies report little change or even a decrease in ChREBPα with high-carbohydrate diets [84,86,94,95]. The data related to ChREBPα from this study, along with those reported in the literature, indicate that its variations are not significant due to obesity or metabolic complications; even the MUL group exhibited a modest decrease in fasting individuals. ChREBPβ levels are increased in individuals with metabolic dysfunction, as seen in the MUO group, particularly in the MUL group. Despite this, it is not significant, as corroborated by the positive correlation between the HOMA IR index and liver ChREBPβ mRNA expression in this study. Liver ChREBPβ mRNA and protein expression, in both mice and humans, is robustly increased by high-carbohydrate diets and obesity, often by 10- to 20-fold, compared to metabolically healthy controls. This upregulation is strongly linked to increased dietary carbohydrate intake and is associated with enhanced expression of lipogenic genes as well as a greater risk of hepatic steatosis [86,94,95,96]. This is consistent with findings linking ChREBPβ to more advanced liver damage [5].

However, a study on total ChREBPβ knockout mice shows that the loss of ChREBPβ alone has modest effects on hepatic gene expression and metabolic adaptation, suggesting that ChREBPα is sufficient to maintain most liver metabolic functions [81]. At the same time, it is challenging to interpret the literature on the inhibition or inactivation of ChREBPα or -β, whether partial in the liver or complete in the body as a whole, in animal models. However, in general, the researchers indicate that they can discriminate between different metabolic phenotypes [85,97]. In this way, we support the observations of Benhamed F et al., who, without differentiating the ChREBP isoforms, concluded that ChREBP could dissociate the presence of steatosis and insulin resistance [98]. In the present study, liver ChREBPα and -β present a differential pattern of metabolic states, in agreement with those mentioned above.

The findings of this study suggest that liver ChREBPα is linked to increased steatosis, as it positively correlates with ALT and liver PPARα expression. Conversely, liver ChREBPβ is associated with insulin resistance, as it exhibits a positive correlation with the HOMA-IR index. This is consistent with some studies that implicate ChREBPβ as a more significant contributor to insulin resistance and the development of T2D [99]. These observations and implications regarding the differentiation of metabolic conditions between the two ChREBP isoforms will require further research to verify.

The transcription factor ChREBPα is the isoform involved in the basal response of lipogenesis, closely regulated by glucose levels. It functions as a metabolic switch, initiating the reaction by translocating to the nucleus when the presence of carbohydrates increases. Otherwise, the binding of 14-3-3 proteins to the low-glucose inhibitory domain (LID) sequesters ChREBPα in the cytoplasm. Still, it is also regulated by phosphorylation, acetylation, and O-GlcNAcylation, influencing its stability and transcriptional activity [82,83,84,85,100]. In contrast, ChREBPβ lacks the LID domain, making it predominantly nuclear-localized and constitutively active. This results in a much stronger and more prolonged lipogenic response, enabling robust metabolic reactions to high carbohydrate levels in the liver once induced by ChREBPα [81,82]. Furthermore, ChREBPβ can downregulate ChREBPα through a negative feedback loop, which fine-tunes glucose-induced gene expression. This process helps to prevent overactivation and maintains metabolic balance in hepatocytes and pancreatic β cells [85,101,102]. The above could explain why an inverse trend is observed between the two isoforms in the heatmap of the liver across the entire sample in this study (Figure 4). While ChREBPα levels remain at the average, +0.5, ChREBPβ increases to +1.5 of the average, aligning with the positive correlation between both isoforms found in this study. When ChREBPβ levels are close to −0.5, they are accompanied by increased ChREBPα levels of +1.5. The same tendency is observed between the groups in the expression regulation in the liver, as shown in Figure 1a, which is in agreement with the regulation between the two isoforms.

### 3.3. FGF21 Expression

*FGF21* is considered a hepatokine, first identified in rodents [103]. It has also been reported in adipose tissue [104,105], although its expression in human adipose tissue has yielded contradictory results. Mraz et al. identified significantly higher expression in the visceral adipose tissue of obese individuals [106]. In contrast, other studies reported very low or even absent expression in both types of adipose tissue, with Ct values greater than 33 or no amplification, in both obese and lean individuals [107,108,109]. Those studies support the finding above.

ChREBP, especially the isoform ChREBPβ [82,110,111,112], is directly related to the increase in *FGF21* expression in the liver, since *FGF21* contains a carbohydrate response element in its promoter where the products of the *MLXIPL* gene bind [7,83,110,111]. Our data support the previous findings, showing a positive correlation between liver ChREBPβ and *FGF21*. A similar pattern of expression is illustrated in the heatmap of Figure 4, where both genes cluster in Branch 3E.

Recent evidence suggests that ChREBP can also regulate *FGF21* indirectly by modulating PTEN, a phosphatase that affects the release of *FGF21*. ChREBP depletion reduces PTEN [113], which in turn increases *FGF21* secretion and improves systemic insulin sensitivity. PTEN has also been linked to the MUL phenotype [114]. Loss of *FGF21* blunts the beneficial metabolic effects of ChREBP depletion or overexpression, confirming its essential role in mediating the systemic actions of hepatic ChREBP [82,113].

On the other hand, the tendency towards increased expression of *FGF21* in both MUL and MUO individuals, as well as its lack of correlation with the HOMA-IR index, is noted. The above suggests that *FGF21* may function as an adaptive response to metabolic alterations induced by liver ChREBPβ [82]. Remarkably, the increased expression of ChREBPβ and *FGF21* in the liver is what most effectively distinguishes metabolically healthy individuals from those with unhealthy states, regardless of the presence of obesity [82]. In our study, liver *FGF21* expression and serum GGT levels are markers in the MUL group. *FGF21* has been suggested as a marker of mitochondrial adaptive flexibility [115], mitochondrial dysfunction [116,117], autophagy deficiency [118], and endoplasmic reticulum stress. Previous data have identified an increase in mRNA *FGF21* expression associated with hepatic steatosis in humans [119]. However, a comprehensive stratification of the entire spectrum of MASLD in relation to *FGF21* expression is needed [120]. Therefore, the data from this study aid in elucidating the above.

The hepatokine *FGF21* is a member of the fibroblast growth factor family that enhances insulin sensitivity, decreases hepatic lipid accumulation, and increases energy expenditure in peripheral tissues, such as adipose tissue [121,122,123]. In animal models, FGF21 has demonstrated beneficial effects on liver fat accumulation, inflammation, and fibrosis. FGF21 analogs, such as Efruxifermin and Pegbelfermin, have shown efficacy in reducing liver fat and improving fibrosis in clinical trials [124,125,126]. FGF21 analogs are currently in Phase II and III clinical trials but have not yet received regulatory approval [80,127,128].

The regulation of *FGF21* by ChREBP in response to glucose depends on the presence of functional PPARα in the liver [7,83,129]. This finding is evident from rescue experiments in which re-expressing active ChREBP in ChREBP-deficient mice successfully restores *FGF21* production. However, this restoration is contingent upon the presence of PPARα, underscoring the necessity of both factors to achieve full *FGF21* induction by glucose [7]. Additionally, PPARα is a well-established direct regulator of *FGF21*, particularly during fasting and in response to fatty acids [7], which supports lipid oxidation and ketone production [130,131]. The above explains the positive correlation between PPARα and *FGF21* in our study. The upregulated expression and secretion of *FGF21* may imply that obesity could be a condition resistant to *FGF21* [132]. In the MUO group, both ChREBP and PPARα converge to regulate *FGF21* expression.

### 3.4. Study Limitations

The main limitation of this descriptive cross-sectional study is the inability to demonstrate causal effects. Additionally, the sample is compromised due to the overrepresentation of women. Furthermore, the recruitment process predominantly included individuals who had undergone bariatric surgery or cholecystectomy to obtain a biopsy, alongside presenting comorbidities associated with MetS, and were at high risk of developing cardiovascular disease.

Identifying subgroups such as MHO and MUL individuals represents a considerable challenge in this type of sample. Therefore, the reduced sample size bias is present, which increases the likelihood of false negatives, implying a greater possibility of not detecting significant differences between groups, even when they exist. To overcome the limitations above, we calculated the statistical power based on the effect size and validated our statistical results using small samples.

The results obtained regarding the statistical power of each group, tissue, and gene expression indicate that, solely in the liver, *PPARA* showed a power close to 100%, and the MUL group for ChREBPβ obtained an adequate statistical power (1-β = 0.84); however, the statistical power to detect differences in *FGF21* expression is low when comparing control subjects to all groups, especially for MUL (1-β = 0.16). In OAT, none of the genes in the different groups reached sufficient statistical power. On the other hand, in SAT, the MUO group for ChREBPα achieved a power of 1-β = 0.9, while both MUL and MHO obtained good levels for ChREBPβ (1-β = 0.81 and 1-β = 0.97, respectively). However, the statistical power for PPARA was low across all groups in SAT. The above suggests that the lack of significance of the data on *FGF-21* in the liver and ChREBPβ gene expression in OAT may stem from the limited number of participants in the subgroups. Accordingly, these findings require replication in independent cohorts to confirm their validity and generalizability across populations with diverse genetic backgrounds.

## 4. Materials and Methods

### 4.1. Subjects

This cross-sectional analytical study included 55 patients undergoing elective cholecystectomy or bariatric surgery. The patients were classified according to the definition of metabolically healthy obesity by Smith et al., into four groups: metabolically healthy obese (MHO, n = 8), metabolically unhealthy obese (MUO, n = 29), metabolically unhealthy lean (MUL, n = 6), and metabolically healthy lean (MHL, n = 12) as a control group. This classification differentiates MHO, characterized by the presence of obesity without the adverse metabolic effects commonly associated with excess body fat, defined by having ≤2 components of metabolic syndrome and normal HOMA-IR (Homeostatic Model Assessment of Insulin Resistance). Criteria to define MHO include the following: (a) absence of diagnosis or therapy of cardiometabolic disease as prediabetes, type 2 diabetes (T2D), hypertension, dyslipidemia, MASLD, chronic kidney disease (CKD) or/and cardiovascular disease (CVD); treatment with blood pressure, lipid, or diabetes medications; (b) healthy cardiometabolic profile defined as fasting TG < 95 mg/dL; HDL-C ≥ 40 mg/dL in men and ≥50 mg/dL in women; fasting glucose < 100 mg/dL; blood pressure < 130/85 mmHg; and plasma C-reactive protein concentration < 3 mg/L, in addition to different metabolic syndrome components with or without criteria for HOMA-IR and LDL cholesterol [4]. In contrast, MUO is marked by the presence of at least ≥ 2components of metabolic syndrome and/or insulin resistance, increasing the risk of cardiovascular diseases and other metabolic complications [4]. Individuals with a body mass index (BMI) ≤ 25 were grouped according to the same criterion, metabolically healthy and unhealthy, as MUL and MHL. Stratified sampling was carried out to ensure homogeneity between groups categorized by age and sex.

Each participant underwent simultaneous biopsies of liver tissue (LIVER), omental adipose tissue (OAT), and subcutaneous adipose tissue (SAT). Exclusion criteria included the following: incomplete data, the presence of acute liver disease, cholecystitis, pancreatitis, or cirrhosis (as determined by histopathological assessment), hypothyroidism, cancer, infections, autoimmune diseases, psychiatric disorders, addictions, and women who were undergoing hormone replacement therapy, in menopause, or pregnant. All participants signed informed consent, and approval was granted by the bioethics and research committees of the Hospital Civil Nuevo “Juan I. Menchaca” (No. 00122) and COFEPRIS registration 17CI14039116, in accordance with the guidelines of the 75th WMA General Assembly held in Helsinki, Finland, in October 2024 [133].

### 4.2. Anthropometric Measurements

Body weight and body composition were determined using the Body Composition Analyzer and Goal Setter (Tanita Company, Tokyo, Japan; model TBF-300A). BMI was calculated by the formula weight (kg)/height (m^2^). Waist (the narrowest diameter between the lowest borders of the rib cage and the iliac crest) and hip (the widest portion of the buttocks) circumference measurements using a Lufkin Rosscraft^®^ tape (Lufkin Rosscraft^®^, Houston, TX, USA; model W606) allowed calculation of the waist–hip ratio (WHR).

### 4.3. Biochemical Measurements

Fasting blood samples were collected from patients, and serum aliquots were stored at −80 °C until analysis to preserve their integrity. The following biochemical assays were performed simultaneously to minimize analytical variability, using a dry chemistry system (Vitros 250 Analyzer, Ortho Clinical Diagnostics, Johnson & Johnson Co., Rochester, NY, USA): alanine aminotransferase (ALT), aspartate aminotransferase (AST), gamma-glutamyl transferase (GGT), glucose, triglycerides (TG), total cholesterol (TC), and high-density lipoprotein cholesterol (HDL-c). Low-density lipoprotein cholesterol (LDL-c) was calculated according to Friedewald’s formula [134]. Very-low-density lipoprotein cholesterol (VLDL-c) was calculated as total cholesterol minus (LDL-c + HDL-c).

Insulin levels were measured using the LIAISON^®^ Insulin Immunoassay Analyzer (DiaSorin, Saluggia, Italy). The minimum dose that the test can detect is 0.17 mIU/mL. Insulin resistance (IR) was calculated using the homeostatic model assessment (HOMA-IR) index as follows: HOMA-IR index = (fasting insulin [μIU/mL] × fasting glucose [mg/dL])/405. A HOMA-IR index cut-off ≥ 2.5 was considered as the indicator of IR [135].

The C-reactive protein was determined by an hs-CRP immunofluorescence assay test kit and detected by a Getein 1100 Biotech Quantitative Analyzer (Getein Biotech Inc., Nanjing, China). The test detection ranges reported are 0.5 m–200 mg/L, and the cut-off value is <3 mg/L.

### 4.4. Tissue Samples, RNA Extraction, and Real-Time qPCR 

OAT and SAT biopsies were taken from the omental and superficial epigastric/umbilical regions, respectively, while the liver biopsy was taken from the left hepatic lobe. Tissue samples were stored at −80 °C. Total RNA was extracted using TRIzol^®^ solution (Invitrogen, Carlsbad, CA, USA) and performed as previously described [136]. RNA quantity and purity were estimated using a Nanophotometer P-Class (Implen Inc., München, Germany). The integrity of samples was tested by 1% agarose gel electrophoresis.

In total, 1 µg of total RNA was reverse-transcribed using a High-Capacity cDNA Reverse Transcription Kit (Applied Biosystems™, Carlsbad, CA, USA). The obtained cDNA was stored at −20 °C until use. According to the manufacturer’s instructions, all qPCR assays were performed in a LightCycler^®^ 96 Real-Time PCR System (Roche Diagnostics, Mannheim, Germany). The mixture and thermal cycling conditions in the PCR reaction, using 2 µL of cDNA, have been previously described [137].

The assays were reproducible when conducted in triplicate or quadruplicate, yielding comparable results. The primers and probes used were from Taqman^®^ Gene Expression (Applied Biosystems™, Carlsbad, CA, USA) for the following genes: *PPARA* (ID: Hs00947539_m1), *FGF21* (ID: Hs00173927_m1), *MLXIPL* isoform ChREBPα (ID: Hs00263027_m1), and *MLXIPL* isoform ChREBPβ of our own design. A custom TaqMan probe design was necessary for the ChREBPβ isoform, as no pre-designed probes were available. Using primers reported by Ramírez-Meza et al. (2019), a sequence was generated that included exon 1b spliced with exon 2, bypassing exon 1a while retaining other exons of ChREBPα [136]. The design process involved using Integrated DNA Technologies (IDT) tools to generate five potential assay designs, incorporating start and end sites for probes and primers, as well as extension, melting temperature, and GC content. The final criteria were selected using Thermofisher Scientific resources. The housekeeping gene was the RNA polymerase II subunit (*POLR2A*, ID: Hs00172187_m1). The data were normalized to the expression level of MHL control subjects, and the relative gene expression was estimated according to the 2^−ΔΔCt^ method [138].

### 4.5. Liver Histology

The liver biopsy was placed into 1 mL of paraformaldehyde for fixation. Hematoxylin–eosin staining was performed using standard protocols with histopathological evaluation by an expert pathologist, based on the system established by the Pathology Committee of the NASH Clinical Research Network. The study groups were stratified according to Metabolic Dysfunction-Associated Steatotic Liver Disease [2], using a parameter of more than 5% affected hepatocytes [139].

### 4.6. Statistical Analysis

Quantitative data were expressed as the mean ± standard error of the mean (SEM) and qualitative data as percentages. The chi-square test (X^2^) was used to analyze the qualitative variables. To evaluate data distribution, a Shapiro–Wilk test was applied to determine whether the variables followed a normal distribution; then, as appropriate, we applied either ANOVA or the Kruskal–Wallis test. These analyses were followed by post hoc Bonferroni and Dunn tests to identify specific intergroup differences while controlling for multiple comparisons. Spearman’s correlation coefficient was used to assess associations between parameters. Data analyses were performed using R Studio v.4.2.3 and GraphPad Prism 8.0.1. Statistical significance was set at *p* < 0.05. Normalized gene expression values (relative to MHL controls) were visualized through a heatmap to illustrate clustering relationships between genes and study groups, generated with ClustVis 2.0: graphic software for visualizing 2D density distributions [140]. This method identifies patterns and co-regulated genes that could share functional pathways. By applying hierarchical clustering, genes and samples are grouped by expression similarity, generating dendrograms that visualize their relatedness and enable an integrated understanding of transcriptional relationships across different experimental conditions. Sample size and power calculations were set with a significance level (*p*-value) of <0.05 and power at 1-β = 0.80, using a total of 60 individuals and 15 per group, based on the differential liver expression of *PPARA* [68]. To calculate post hoc statistical power for the expression analyses, it is essential to determine the effect size (Cohen’s d); this measure quantifies the magnitude of the difference between two means, independent of sample size. It provides an estimate of the practical or biological relevance of the results, complementing the *p*-value by indicating whether a statistically significant difference is also meaningful in real terms.

## 5. Conclusions

**Figure 5:**
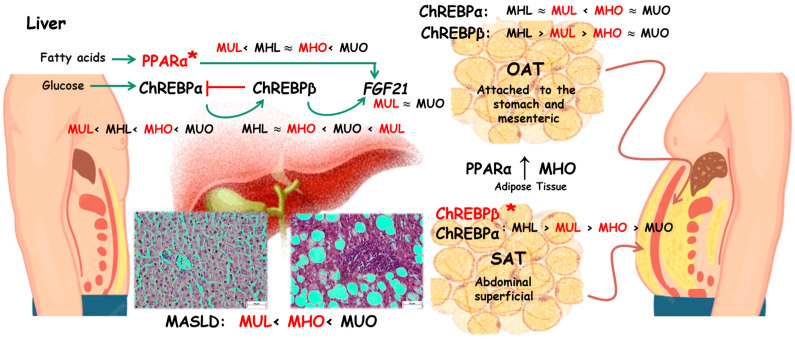
MASLD presence and mRNA expression by tissue-type-specific pattern of Fibroblast Growth Factor 21 (FGF21); Peroxisome Proliferator-Activated Receptor Alpha (PPARα), which regulates lipid metabolism and energy expenditure; and Carbohydrate-Responsive Element-Binding Protein (isoforms ChREBPα and -β) in metabolically healthy lean (MHL), metabolically unhealthy lean (MUL), metabolically healthy obese (MHO), and metabolically unhealthy obese (MUO) conditions. * Statistically significant (*p* < 0.05). Subcutaneous (SAT) and omental adipose tissue (OAT). Scale bar = 50 μm.

The findings of this research are relevant due to the unprecedented study of gene interactions in simultaneous biopsies of tissues in humans in MHO and MUL individuals. Conducting the study on human samples lends the findings high clinical applicability and relevance, as these genes are therapeutic targets in obesity and its complications. A tissue-specific expression pattern was observed: PPARα differential expression was seen in liver tissue, while ChREBPβ changed in SAT, reflecting distinct metabolic adaptations between the study groups. Correlation analyses revealed that hepatic ChREBPβ expression was positively associated with insulin resistance and FGF21, suggesting coordinated regulation under metabolic stress. Conversely, OAT ChREBPβ was negatively correlated with body fat percentage, body weight, and serum insulin levels, indicating an adaptative effect. Finally, increased FGF21 expression in MUL and MUO groups may aid as a metabolic biomarker of impaired energy homeostasis and compensatory hepatic response (Figure 5).

## Figures and Tables

**Table 1:** Anthropometric, biochemical, and histopathological analyses of the study groups.

Groups	MHL	MUL	MHO	MUO
	n = 12	n = 6	n = 8	n = 29
Male/Female n (%)	2(17)/10(83)	0/6(100)	1(13)/7(87)	4 (18)/25(86)
Age (years)	42.8 ± 3.5	37.3 ± 6.5	34.3 ± 2.6	34.2 ± 1.6
Weight (kg)	61.8 ± 2.3	61.5 ± 2.4	102.4 ± 4.9 ^b^	106.5 ± 4.0 ^c^
BMI (kg/m^2^)	24.1 ± 0.6	24.1 ± 1.1	38.9 ± 1.4 ^b^	40.0 ± 1.3 ^c^
Waist circumference (cm)	80.2 ± 1.8	78.1 ± 1.1	114.0 ± 4.0 ^b^	114.6 ± 3.0 ^c^
Body fat (%)	29.0 ± 2.0	32.3 ± 2.0	48.1 ± 1.7 ^b^	46.2 ± 1.2 ^c^
Glucose (mg/dL)	87.5 ± 4.4	87.2 ± 7.4	74.3 ± 5.2	83.4 ± 3.0
Insulin (mIU/mL)	4.8 ± 0.7	11.2 ± 2.5 ^a^	7.8 ± 1.5 ^b^	19.6 ± 1.8 ^c,d^
HOMA-IR	1.1 ± 0.2	2.5 ± 0.7	1.4 ± 0.2	4.2 ± 0.5 ^c,d^
hs-CRP (mg/L)	3.8 ± 1.2	2.5 ± 0.3	5.9 ± 1.3	5.3 ± 0.5
Cholesterol (mg/dL)	195.2 ± 14.0	213.2 ± 15.6	149.3 ± 7.1	165.2 ± 6.1
TG (mg/dL)	119.6 ± 15.3	170.4 ± 36.4	108.4 ± 13.7	164.7 ± 13.2
LDL-c (mg/dL)	100.3 ± 16.1	136.0 ± 14.2	85.7 ± 7.1	94.0 ± 5.5
HDL-c (mg/dL)	53.0 ± 4.0	43.0 ± 4.2	41.9 ± 4.1	38.4 ± 1.8
VLDL-c (mg/dL)	24.0 ± 3.1	34.0 ± 7.3	21.7 ± 2.5	32.9 ± 2.6
ALT (U/L)	23.5 ± 3.5	45.8 ± 27.7	22.0 ± 2.3	41.5 ± 6.1 ^d^
AST (U/L)	34.8 ± 6.3	43.8 ± 9.4	27.0 ± 1.2	39.2 ± 3.2 ^d^
GGT (U/L)	26.0 ± 6.0	40.5 ± 15.4	24.4 ± 1.9	39.8 ± 6.6
MASLD n (%)	0	2 (33.3)	5 (62.5) ^b^	25 (85.2) ^c,e^

Clinical, anthropometric, and biochemical characteristics of the study groups: metabolically healthy lean (MHL), metabolically unhealthy lean (MUL), metabolically healthy obese (MHO), and metabolically unhealthy obese (MUO). Quantitative data are presented as the mean ± SEM, and qualitative variables are expressed as percentages. Sex distribution did not differ significantly between groups (χ^2^ = 29.5, Fisher’s exact test, *p* > 0.05). Superscript letters indicate significant differences (*p* < 0.05) between groups as follows: ^a^ MHL vs. MUL, ^b^ MHL vs. MHO, ^c^ MHL vs. MUO, ^d^ MHO vs. MUO, ^e^ MUO vs. MUL. BMI: Body mass index. HOMA-IR: Homeostasis model assessment of insulin resistance. hs-CRP: High-sensitivity C-reactive protein. TG: Triglycerides. LDL-c: Low-density lipoprotein cholesterol. HDL-c: High-density lipoprotein cholesterol. VLDL-c: Very-low-density lipoprotein cholesterol. ALT: Alanine aminotransferase. AST: Aspartate aminotransferase. GGT: Gamma-glutamyl transpeptidase. MASLD: Metabolic Dysfunction-Associated Steatotic Liver Disease.

## Data Availability

The original contributions presented in this study are included in the article. Further inquiries can be directed to the corresponding author.

## Abbreviations

The following abbreviations are used in this manuscript:

ALT	Alanine Aminotransferase
AST	Aspartate Aminotransferase
BMI	Body Mass Index
ChREBP	Carbohydrate Response Element Binding Protein
CKD	Chronic Kidney Disease
CVD	Cardiovascular Disease
GGT	Gamma-Glutamyl Transpeptidase
FLI	Fatty Liver Index
FGF21	Fibroblast Growth Factor
HDL-c	High-Density Lipoprotein Cholesterol
HOMA-IR	Homeostasis Model Assessment of Insulin Resistance
hs-CRP	High-Sensitivity C Reactive Protein
LDL-c	Low-Density Lipoprotein Cholesterol
MASLD	Metabolic Dysfunction-Associated Steatotic Liver Disease
MetS	Metabolic Syndrome
MHL	Metabolically Healthy Lean
MHO	Metabolically Healthy Obese
MLXIPL	MLX Interacting Protein-Like
MUL	Metabolically Unhealthy Lean
MUO	Metabolically Unhealthy Obese
OAT	Omental Adipose Tissue
PPARA	Peroxisome Proliferator-Activated Receptor Alpha
SAT	Subcutaneous Adipose Tissue
TG	Triglycerides
T2D	Type 2 Diabetes
VAI	Visceral Adiposity Index
VLDL-c	Very-Low-Density Lipoprotein Cholesterol
